# Analysis of distribution method of designed air quantity in coal mine ventilation—a case study

**DOI:** 10.1038/s41598-024-61787-9

**Published:** 2024-05-13

**Authors:** Yongyin Wang, Qizhi Pan, Lin Gao, Yunqin Cao, Ping Liu, Hanhua Yi, Changsi Gao

**Affiliations:** 1 Mining College of Guizhou University, Guiyang, 550025 China; 2 National and Local Joint Laboratory of Engineering for Effective Utilization of Regional Mineral Resources from Karst Areas, Guiyang, 550025 China; 3 Coal Mine Roadway Support and Disaster Prevention Engineering Research Center, Beijing, 100083 China; 4 Key Laboratory of Mining Disaster Prevention and Control, Qingdao, 266590 China

**Keywords:** Energy science and technology, Engineering

## Abstract

In a coal mine, air leakage exists in some roadways through doors and other ventilation structures inevitably. Based on this opinion, there are different views on whether these roadways must be assigned airflow in coal mine ventilation design. This paper analyses some relevant regulations and criteria on the designed air quantity of coal mines. Then, based on the ventilation design of the Guizhou Yizhong Coal Mine, through the study of the calculation of needed air quantity of every working place and its distribution method in coal mine ventilation design, this paper puts forward that explosion-proof door, safety exit, and other short distance roadways with ventilation structures need not assign airflow in coal mine ventilation design, while some long-distance roadways need. Additionally, it presents the main reason to support this opinion, gives the distribution method of inner air leakage quantity, which comes with the calculation of the designed mine total air quantity, puts forward the remedy method for the air leakage through ventilation structures in a coal mine ventilation system, then offers the mine operator with the basic opinions for the day-to-day planning and effective operation of a coal mine ventilation system.

## Introduction

Safety Regulations of Coal Mine (China)^1^ and other regulations and criteria^2–7^ all stipulate the calculation method of the designed mine total air quantity and its distribution method.

The calculation method of air quantity is as follows^1–5,7–10^.

Firstly, calculate the needed air quantity according to the maximum number of workers who work underground simultaneously. Secondly, calculate the required air quantity of each working face, developing face, chamber, and other roadways which need airflow according to gas emission rate, air absorption volume of the auxiliary fans, explosive consumption, and others. Especially, the air quantity of other roadways in a newly designed coal mine can be calculated as 3–5% of the total air quantity of the calculated working face, developing face, and chambers. Thirdly, the maximum value of the first two steps multiplied by the mine ventilation coefficient is the total mine air quantity of the designed coal mine. Mine ventilation coefficient includes the inner air leakage and the uneven air quantity distribution within the mine. It is a comprehensive index reflecting the underground ventilation structures and ventilation management level.

The air distribution method on needs in coal mine design is as follows^1–5,7–10^.

Firstly, distribute airflow to every working face, developing face, chamber, and other roadways according to calculated air quantity. The remaining airflow is allocated to other air-demand sites according to a certain proportion to ensure the safety of pedestrians and roadways.

It’s essential to calculate the required air quantity and to distribute airflow on needs in underground coal mine ventilation design, because the calculation of mine ventilation resistance is based on the distributed airflow and the coefficient of frictional resistance of the mine.

The main fan is selected according to the calculated mine ventilation resistance and the required air quantity, which further affects mine production, mine safety, fan efficiency, and electricity consumption^11–14^.

## Different opinions about the distribution of designed total mine air quantity

A coal mine ventilation system consists of interconnected roadways, working locations, chambers, and ventilation structures. According to airflow distribution method on needs, the designed total mine air quantity must distribute to every air-demand location.

There are two different understandings of air-demand locations, especially for some roadways with ventilation structures. A focus of the discussion was on whether these roadways with ventilation structures need to be assigned airflow in mine ventilation design. The traditional distribution method of designed air quantity adheres that due to air leakage existing through ventilation structures, all underground roadways with ventilation structures, including explosion-proof doors, safety exits, short cross-cut, and others, all need to be distributed with airflow^1–3,5^. According to this opinion, not only the working face, developing face, and chamber need to be distributed with airflow; other roadways with ventilation structures also need to be distributed with airflow. The new opinion on distribution method of designed air quantity adheres that most of the underground roadways with ventilation structures, including explosion-proof doors, safety exits, short cross-cut, and others, do not need to be distributed with airflow^1–3,5,11^; if some of them need to be distributed with airflow should set up regulators. According to the different opinions of the air-needed locations, there are considerable differences in the calculated air quantity requirement and the distribution method of air quantity, which further influence the selection of main fan and fan efficiency, and finally affect mine production, mine safety, and the power consumption of coal mine ventilation.

The suitable mine design of ventilation plays a vital role in mine productivity, profit, and safety. Purpose of this paper is to analyze which roadway is required to be distributed with air quantity, put forward a reasonable method to distribute air quantity in coal mine ventilation design, and then provide the mine operator with the essential opinions and effective operations for day-to-day management in a coal mine ventilation system.

## Literature survey

Currently, many regulations and criteria of coal mine stipulate the calculation and distribution method of mine air quantity. For example, the Safety Regulations of Coal Mine^1^ provide the calculation method of air quantity, the distribution method of air quantity in coal mine design, and the acceptable maximum and minimum air velocity of each roadway and working place. The other regulations and criteria^2–5^ stipulate the calculation and distribution methods of mine air quantity, provide technical requirements about air quantity and air velocity also. Professional technical books^8,9,15^ about coal mines provided mine air quantity calculation methods and distribution methods also. However, these regulations, criteria, and professional books are not clearly stated which roadway needs airflow in coal mine design, and which need not, especially for short-distance roadways with ventilation structures.

Ventilation system analysis for underground coal mines has remained mostly unchanged since the Atkinson method was made famous by McElroy in 1935^10,16^. Data available to ventilation technicians and engineers are typically limited to in-situ^10,11,16,17^. Thus, there are few papers on mine ventilation design, except for some papers on mine ventilation safety. For example, Wang et al.^11,18^ analyzed the distribution method of designed air quantity in mine ventilation design. They provided the idea that some-short distance roadways need not be distributed with airflow, but didn’t analyze it in detail to support this idea. Watson et al. and Hartman et al.^16,19^ presented a new technique for estimating underground drift friction factors that work by processing 3D point cloud data obtained by a mobile LiDAR. Develo et al.^20^ optimized the ventilation system of a zinc mine by replacing the existing western orefield fans with more giant fans from the inactive southern orefield workings to increase the airflow of the western orefield and to reduce the ventilation cost according to simulation results of the Wentsim™ software. Pach et al.^21^ introduced a new method for lowering ventilation costs based on an algorithm that allows the determination of the resistance of stopping, the head of the fans, and the air quantity for which air distribution is optimal. As a result, the total power output of the fans is at the lowest level, which yields a reduction in ventilation costs using this new method. To reduce the operating expenses of ventilating and cooling underground mines permanently, Du Plessis et al.^22^ discussed some strategies to reduce energy consumption by using optimizing cooling and ventilation network simulation models. Heriyadi et al.^23^ examined the importance of evaluating and analyzing the need for ventilation systems in underground coal mines with a case study of several mines operating in Sawahlunto. These papers mainly focus on mine friction factors and ventilation costs, rarely discuss the calculation and distribution of mine air quantity during coal mine design. Particularly, they didn’t give further discussion of whether these roadways with ventilation structures need to be distributed with airflow, such as short crossheading, ventilation bypass, and so on.

## A case study: ventilation design of Guizhou Yizhong Coal Mine

### Overview of the safety conditions of Guizhou Yizhong Coal Mine

The designed mine capacity of Guizhou Yizhong Coal Mine is 0.6 Mt/a. Coal seams in the minefield are roughly in east–west direction with strike length of 4.5 km, incline width of 2.5 km in the west, and 1.2 km in the east. Coal seams No.9, No.11, No.12, No.14, and No.17 are minable with spacing ranging from 2 to 44 m. The thickness of minable coal seams varies from 0.81 to 3.18 m. The Guizhou Yizhong Coal Mine is mining the close spacing coal seam group, mainly thin and medium thick coal seams in which coal dust is explosive, and coal seam is not easy to spontaneous combustion. It’s a coal (rock) and gas outburst mine.

### Overview of the mine design of Guizhou Yizhong Coal Mine

The design of the Yizhong Coal Mine adopts inclined shaft development, equipped with a main inclined shaft, a service inclined shaft, and an air-return inclined shaft. The main inclined shaft adopts a large dip angle belt conveyor to transport coal and also serves as an intake airway. The service inclined shaft is used for pedestrian, material transport, gangue transport, water supply pipeline laying, drainage pipeline laying, compressed air pipeline laying, and also services as an intake airway. The air-return inclined shaft is used for air-return roadway and gas drainage pipeline laying. The air-return inclined shaft is equipped with two air ducts and a safety exit near the ground.

The minefield is divided into a mining level. The horizontal elevation of the mining level is + 1400 m. Above + 1400 m in the west of the minefield is named 11 rise district. Below + 1400 m in the west of the minefield is designated as 21 dip district. The east of the minefield is quoted as 12 dip district. The district rise or district dip is located in the floor of No.17. It adopts cross-cuts to contact each coal seam. The first mining district is 11 rise district using the downward mining method between sections and between coal seams in the same section. Coal seam No.9 is the first mined coal seam, which thickness varies from 0.59 to 2.88 m.

There is one fully-mechanized longwall face, 2 gate developing faces, and one driving place in the floor of No.17 used as a gas drainage laneway for the designed commissioning period of the coal mine. Predicted relative gas emission rate is 93.84 m^3^/t, and the absolute gas emission rate is 118.48 m^3^/min during mining coal seam No.9.

The designed ventilation system is a centralized exhausting system. The working face, developing face, and district substation adopt separate airflow. The working face adopts a "U" ventilation system. The developing face is equipped with auxiliary fans using a blowing ventilation system.

**Figure 1:**
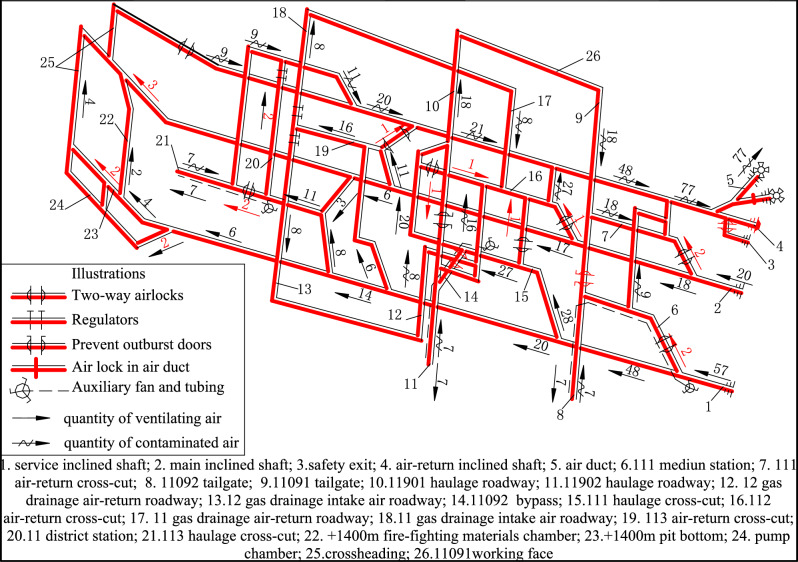
Roadway layout and air distribution of the designed commissioning period of 11 district.

The roadway layout of the designed commissioning period is shown in Fig. 1.

According to the ventilation design of the Guizhou Yizhong Coal Mine, the calculated air quantity of the designed commissioning period is shown in Table 1.

**Table 1:** The calculated air quantity and air distribution for the designed commissioning period.

No	Location	Required air quantity (m^3^/s)	Cross-sectional area (m^2^)	Designed air velocity (m/s)
1	Working face	18	5.4	3.3
2	Gate developing face 1	7	10	0.7
3	Gate developing face 2	7	12	0.58
4	Driving face	7	10	0.7
5	District substation	2	12	0.17
6	Pump chamber	2	12	0.17
7	+ 1400 m fire-fighting materials chamber	2	12	0.17
8	11 gas drainage roadway	8	6.4	1.25
9	12 gas drainage roadway	8	6.4	1.25
10	Other locations	3		
11	Total (multiplied by mine ventilation coefficient)	77		

The total air quantity of the working face, gate developing faces, driving face, gas drainage roadways, and chambers is 61 m^3^/s. According to Zhang ^5^, the air requirement of other locations shall be calculated by a factor of 3–5% of the total of working face, developing face, and chamber in the ventilation design of a new mine. The calculated air quantity of other locations is 3.0 m^3^/s. If the central ventilation system is adopted, the mine ventilation coefficient is between 1.2 and 1.25. Thus, the total air quantity of the mine is 77 m^3^/s, increased by 13 m^3^/s. In other words, after considering other roadways and the mine ventilation coefficient, the total mine air quantity is increased by 16 m^3^/s.

### The characteristic of the design for air distribution

The designed air distribution is based on the opinion that all underground roadways must be distributed with airflow. The distribution of air quantity for every working place and chamber is shown in Table 1 and Fig. 1. The distribution of the increased airflow after considering other roadways and the mine ventilation coefficient is shown in Table 2 and Fig. 1.

**Table 2:** Air distribution of other air-demand sites for the designed commissioning period.

No	Location	Distributed airflow (m^3^/s)	Cross-sectional area (m^2^)	Air velocity (m/s)	Remark
1	111 air-return cross-cut	2	11.9	0.17	
2	111 medium station	2	11.9	0.17	
3	112 air-return cross-cut	1	11.9	0.08	Near the main inclined shaft
4	Bypass	1	7.7	0.13	Between 111 haulage cross-cut and 112 air-return cross-cut
5	112 air-return cross-cut	1	11.9	0.08	Near 111 haulage cross-cut bypass
6	11,902 haulage roadway	1	14.2	0.07	
7	113 air-return cross-cut	1	11.9	0.08	
8	113 haulage cross-cut	2	14.2	0.14	
9	+ 1400 pit bottom	2	14.2	0.14	
10	Bottom of the main inclined shaft	3	14.2	0.21	
11	Total	16			

The characteristic of the design for air distribution is as follows:

Firstly, for roadways of the same cross-sectional area with doors, the designed air quantity is different. For example, the cross-sectional area of the 111 medium station and the 112 air-return cross-cut is 11.9 m^2^, but the designed airflow is 2 m^3^/s and 1 m^3^/s, respectively.

Secondly, most air velocity in roadways distributed with air quantity is lower than the acceptable minimum air velocity. For example, the air velocity in 112 air-return cross-cut and 11,902 haulage roadway is lower than 0.15 m/s, which is the allowable minimum air velocity of roadways in rock.

Thirdly, some of the roadways with ventilation structures are not equipped with air quantity, such as the safety exit of the air-return inclined shaft, the explosion-proof door of the air-return inclined shaft, the alternative air duct of the air-return inclined shaft, and the connecting lane between the 11,902 tailgate and 11,901 tailgate. Some of the roadway with flexible tubing through ventilation doors is distributed with air quantity while the others not. For example, the 111 medium station and the 113 haulage cross-cut is distributed with 2 m^3^/s air quantity, while the 11,902 bypass is not distributed with air quantity.

### The problem of the design for air distribution

The main reasons for the design of air quantity distribution of Guizhou Yizhong Coal Mine are as follows: firstly, all underground roadways must be equipped with airflow because air leakage through doors is inevitable. Secondly, the mine ventilation coefficient is taken into account in the calculation of the mine total air quantity. Thus, the increased air quantity must be distributed to each leakage site. Based on this opinion, it caused some questions about the designed air quantity distribution of the Guizhou Yizhong Coal Mine.

The increased air quantity calculated by other locations and mine ventilation coefficient is insufficient for all these different locations and roadways with ventilation structures. The designer of Guizhou Yizhong Coal Mine has to distribute 2 m^3^/s or 1 m^3^/s to some roadways with doors, even some of the roadways are not equipped with air quantity because there is no more air quantity. It is contrary to the opinion that all underground roadways must be distributed with air quantity, especially for some roadways with doors that are not distributed with air quantity.

The air velocity in some roadways with doors will not meet the acceptable minimum air velocity. Regulations^1–3,5^ stipulate that the allowable minimum air velocity of roadway in rock is 0.15 m/s and roadway in coal is 0.25 m/s. According to Table 2, the air velocity of most roadways distributed with air quantity is lower than the allowable minimum air velocity. The reason for this still is no more air quantity for all these roadways. Currently, the cross-sectional area of underground roadways is usually above 12 m^2^ in a fully mechanized coal mine, and the acceptable minimum air quantity is 2 m^3^/s (roadway in rock) or 3 m^3^/s (roadway in coal).

Roadway with doors is distributed with air quantity, which does not meet the tightness requirements of the building standards of doors. According to the building standards of doors, qualified doors should tighten enough to ensure no air leakage.

Based on the analysis above, on the one hand, it can be concluded that the increased air quantity calculated by other roadways and mine ventilation coefficient are insufficient for all roadways with doors; on the other hand, it breaks the tighten requirements of doors if roadway with doors is distributed with air quantity.

## Solutions

In the ventilation design of a coal mine, every working face, developing face, and chamber is distributed with the calculated air quantity, the remaining air quantity shall distribute to each district according to output, number of working faces, number of developing faces, chambers, and other airflow needed roadways according to a certain proportion.

### Auxiliary roadway of the air-return inclined shaft shall not be equipped with air quantity in design

Firstly, the safety exit, the explosion-proof door, and the alternative air duct of the air-return inclined shaft shall not be equipped with air quantity in coal mine design. According to regulations^1–3,5^, the total mine air quantity multiplied by the external air leakage coefficient (k = 1.05–1.15)^1–3,5,7^ is the calculated air quantity of the main fan. In other words, air leakage through the explosion-proof door, safety exit, and alternative air duct are external air leakage, which is calculated in the process of the choice of main fan. So external air leakage can be compensated by the air quantity produced by the external air leakage coefficient in the main fan selection. In addition, not only is the calculated air quantity of main fan larger than the total mine air quantity, but also the main fan’s operating point is higher than the designed point, therefore, the excess air quantity is sufficient to compensate for the external air leakage.

Secondly, referring to the requirements of diffusion ventilation, the safety exit, the explosion-proof door, and the alternative air duct of the air-return inclined shaft shall not be distributed with air quantity. According to regulations^1–3,5^, the requirements for underground workplaces using diffusion ventilation must not be more than 6 m long, the inlet width must not be less than 1.5 m, and where haven’t gas emission. Based on this, it can be concluded that the safety exit, the explosion-proof door, and the alternative air duct of the air-return inclined shaft shall not be distributed with air quantity. Because, on the one hand, these roadways are located near the surface where is no gas emission; on the other hand, these roadways are no more than 6 m long on every side of doors and the inlet width is generally wider than 5 m.

Thus, these roadways shall not be distributed with air quantity in coal mine design.

### Short-distance roadways with doors underground shall not be equipped with air quantity in design

Firstly, referring to the requirements of diffusion ventilation, short-distance roadways with doors shall not be distributed with air quantity. According to regulations^1–3,5^, the requirements for underground workplaces that use diffusion ventilation must not be more than 6 m long, the inlet width must not be less than 1.5 m, and where haven’t gas emission. Based on this, it can be concluded that short-distance roadways with doors should not be distributed with air quantity where is no gas emission and no more than 6 m long on every side of doors. If a short-distance roadway or a long-distance roadway for transportation where need ventilation structures to control airflow does not meet the requirements of diffusion ventilation, the ventilation structure of the roadway should be regulators, rather than doors. In addition, according to regulations^1–3,5,7^, the operator of an underground mine must ensure that sheets or ducts used to direct the ventilation in a working place in any part of the mine are erected and maintained so as to minimize any leakage of air^5,7^.

Secondly, no air leakage is an essential requirement for the construction of air doors. On the one hand, doors with 1–2 m^3^/s or even with more significant air leakage are impossible to construct except for regulators; on the other hand, the basic requirements for the design and construction of doors are no leakage also. Due to the influence of structure and other factors, air leakage through doors or locks exists actually, but it is improper to consciously let doors leakage in coal mine design, though the mine ventilation coefficient (k_m_ = 1.15–1.25)^1–3,5^ has taken into account these factors such as inner air leakage, uneven air distribution, and the management level of the mine. If it is necessary to pass a certain airflow through a long-distance or gas-emission roadway, regulators should be designed to ensure reasonable air quantity and velocity in the roadway.

### The distribution of air quantity caused by mine ventilation coefficient

During the design of a coal mine, if there has gas emission data of a roadway, the needed air quantity of the roadway shall be calculated according to gas emission and meet the requirement of air velocity. In this case, the air quantity caused by mine ventilation coefficient k_m_ = 1.15–1.25 shall allocate to the other roadway according to a certain proportion^1–3,7–9^.

During the design of a coal mine, if there hasn’t gas emission data of a roadway, the needed air quantity of the roadway shall calculate according to 3–5% of the total air quantity of the calculated working faces, developing faces, and chambers. In this case, the air quantity caused by 3–5% of the total air quantity of the calculated working faces, developing faces, and chambers, and caused by mine ventilation coefficient k_m_ = 1.15–1.25 shall distribute to the other roadway according to a certain proportion all^1–3,7–9^.

Usually, regulators should be designed in long-distance or gas-emission roadways to ensure reasonable air quantity and velocity.

### Compensation for air leakage of doors or locks

The air quantity of the main fan is larger than the designed mine total air quantity, which can compensate for air leakage of doors or locks.

Selection of the main fan is as follows^1–3,7–9^.

Firstly, the needed air quantity of the main fan is determined by the equation:

1 $$ {\text{Q}}_{{\text{f}}} = {\text{kQ}}_{{\text{m}}} $$

where k is the external air leakage coefficient of mine (k = 1.05–1.15); Q_m_ is the total mine air quantity (m^3^).

Secondly, the needed static pressure of the axial main fan is determined by the equation:

2 $$ {\text{H}}_{{{\text{sd}}}} = {\text{h}}_{{\text{m}}} + {\text{h}}_{{\text{d}}} \pm {\text{H}}_{{\text{N}}} $$

where h_m_ is the mine total resistance of the ventilation system (Pa), h_d_ is the exit resistance of accessory equipment of the main fan, including fan drift and diffusion tower (Pa), H_N_ is natural air pressure (Pa).

Thirdly, according to the result of Eqs. (1) and (2), a main fan is selected, and the main fan’s designed point is determined. Still, the main fan’s designed point is not possible precisely on one of the actual characteristic curves of the fan chosen, because the main fan’s operating point is determined by the suitable fan blade installation angle and its working resistance.

The working resistance curve is drawn in the characteristic curves of the main fan. The intersection of the active resistance curve with the static pressure curve R_sd_ = H_sd_/Q_f_^2^ of the axial main fan is the main fan’s operating point. The operational air quantity of the main fan can determine according to the main fan’s operating point.

**Figure 2:**
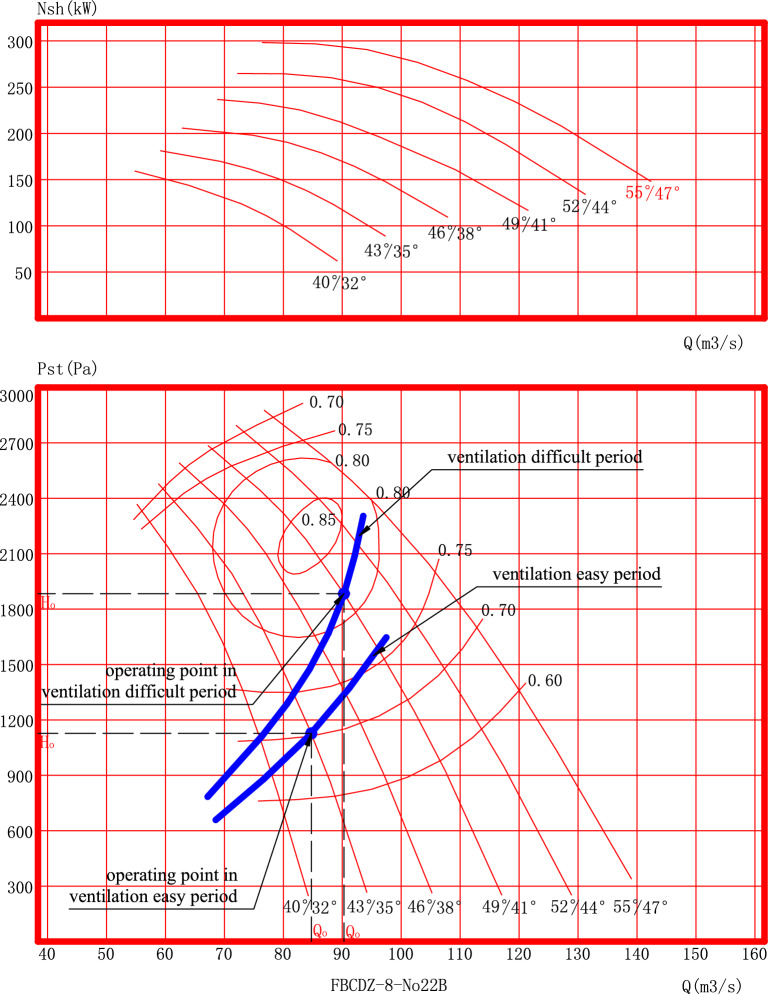
The designed main fan and its operating point of Guizhou Yizhong coal mine.

For example, Table 3 lists the total mine air quantity Q_m_, the needed air quantity of the main fan Q_f_, and the needed static pressure of the axial main fan H_sd_. It is in the designed ventilation easy period and the designed ventilation difficult period of the Guizhou Yizhong coal mine, respectively. According to Q_f_ and H_sd_, the designed main fan is shown in Fig. 2, and its operating point (Q_0_ and H_0_) is shown in Fig. 2 and Table 3.

**Table 3:** Air distribution of other air-demand sites for the designed commissioning period.

No	Q_m_ (m^3^/s)	Q_f_ (m^3^/s)	H_sd_ (Pa)	Q_0_ (m^3^/s)	H_0_ (Pa)	Remark
1	77	80.9	982	84.1	1110	Designed ventilation easy period
2	77	88.2	1849	90.8	1890	Designed ventilation difficult period

According to Fig. 2 and Table 3, it can be seen that the operating air quantity of the main fan is 3.2 m^3^/s larger than the needed air quantity of the main fan in the ventilation easy period, and it is 2.6 m^3^/s larger than the needed air quantity of the main fan in the ventilation difficult period. It also can be seen that the operating air quantity of the main fan is 7.1 m^3^/s larger than the total mine air quantity in the ventilation easy period, and it is 13.8 m^3^/s larger than the total mine air quantity in the ventilation difficult period. The increased air quantity here plus 16 m^3^/s produced by mine ventilation coefficient are sufficient to make up air leakage of various ventilation structures.

Once the main fan is selected, the auxiliary facilities such as the air duct and diffusion tower shall be determined according to the main fan.

### Mine ventilation management can adjust the mine airflow dynamically

The mine ventilation system is a dynamic system according to the status of production. Thus, the operators of mine ventilation management should adjust the air quantity of every air-needed working place effectively. In addition, coal mine safety regulations stipulate^1–3,5^: an airflow measurement system must be established in every coal mine, and a comprehensive air measurement should be conducted once every 10 days. An airflow measurement should be performed when the production status is changed, such as relocation of the working face, developing face, and other air-needed place. Based on this, measures shall carry out to adjust the air quantity of each air-needed place. So, air leakage shall not be considered in the design of coal mine ventilation.

## Comparison between the different distribution methods of designed air quantity

### The different results of distributed air quantity according to the different opinions about the distribution of designed air quantity

The focus of the different distribution methods of designed air quantity was on whether underground roadways with ventilation structures need to be assigned airflow. According to the different opinions, air distribution of these roadways for the designed commissioning period of Yizhong Coal Mine is shown in Table 4.

**Table 4:** Air distribution of different opinions for the designed commissioning period.

No	Location	Cross-sectional area (m^2^)	The traditional distribution method of air quantity		The new distribution method of air quantity		Remark
			Distributed airflow (m^3^/s)	Air velocity (m/s)	Distributed airflow (m^3^/s)	Air velocity (m/s)	
1	111 air-return cross-cut	11.9	2	0.17			Diffusion ventilation
2	111 medium station	11.9	2	0.17	2	0.17	Diffusion ventilation
3	112 air-return cross-cut	11.9	1	0.08			Diffusion ventilation Near the main inclined shaft
4	Bypass	7.7	1	0.13	2	0.26	Between 111haulage cross-cut and 112 air-return cross-cut
5	112 air-return cross-cut	11.9	1	0.08			Diffusion ventilation Near 111 haulage cross-cut bypass
6	11,902 haulage roadway	14.2	1	0.07	3	0.21	
7	113 air-return cross-cut	11.9	1	0.08			Diffusion ventilation
8	113 haulage cross-cut	14.2	2	0.14	3	0.21	
9	+ 1400 pit bottom	14.2	2	0.14	3	0.21	
10	Bottom of the main inclined shaft	14.2	3	0.21	3	0.21	
11	Total		16		16		

According to Table 4, the air velocity of the underground roadways with regulators distributed with air quantity is higher than the allowable minimum air velocity which meets the requirements of Regulations^1–3,5^. While the other short-distance roadways use diffusion ventilation which meets the requirements of Regulations^1–3,5^ also. According to regulations^1–3,5^, the inner air leakage of underground roadways with ventilation structures is included in the mine ventilation coefficient (k_m_ = 1.15–1.25); the external air leakage, such as leakage of air duct is included in the external air leakage coefficient of mine (k = 1.05–1.15); therefore, this new distribution method of designed air quantity meets the requirements of Regulations.

### The different results of the selection of main fan

Due to the different air distribution method, the mine total resistance of the ventilation system (h_m_) is different, thus the needed static pressure (H_sd_) of the axial main fan is different. The selection of main fan according to the traditional technology and the new technology for Yizhong Coal Mine is shown in Table 5.

**Table 5:** The selection of main fan according to different opinions for Yizhong Coal Mine.

No	Item	The traditional technology	The new technology
1	Model of main fan	FBCDZ-8-N0.23B	FBCDZ-8-N0.22B
2	Model of motor	YBFe450S_1_-8	YBFe355S_4_-8
3	Rotating speed (r/min)	740	740
4	Power (kW)	200 × 2	160 × 2
5	Airflow (m^3^/s)	63–141	55–123
6	Pressure (Pa)	820–3125	756–2860
7	Annual power consumption (kWh)	3,504,000	2,803,200

Based on Table 5, it can be concluded that the main fan guided by the new technology has less power consumption.

## Conclusions

Through the theoretical analysis of the distribution method of designed air quantity, and through the practice of ventilation design in Guizhou Yizhong Coal Mine, a reasonable method of air quantity distribution in coal mine ventilation design is provide. Using this new technology, it can reduce the cost of mine ventilation, improve the efficiency of mine ventilation, and reduce the ineffective air leakage of coal mine. In addition, this new technology can provide the mine operator with basic opinions for the day-to-day planning and effective operation of a coal mine ventilation system.

In the design of coal mine ventilation, every working face, developing face, chamber, and roadway with gas emission shall be distributed with the calculated air quantity. The remaining air quantity shall distribute to each district according to output, and the number of working places, then to other air-needed roadways with regulators according to a certain proportion.

The safety exit of the air-return inclined shaft, the explosion-proof door of the air-return inclined shaft, and the alternative air duct shall not be equipped with air quantity in coal mine design. The air leakage of these roadways can be compensated by the air quantity calculated by the external air leakage coefficient of coal mine.

Short-distance roadways shall not be equipped with air quantity in coal mine design, though the internal air leakage is included in the mine ventilation coefficient (k_m_ = 1.15–1.25). To facilitate the construction of ventilation structures with no air leakage, it is not suitable to consciously distribute air leakage for doors and other ventilation structures. The inevitable air leakage in underground coal mines can be compensated by the actual air quantity produced by the main fan.

Mine ventilation management can effectively adjust the air quantity of every air-needed working place, and reduce invalid air leakage.

Regulators shall be constructed in long-distance or gas-emission roadways to ensure reasonable air quantity and air velocity.

## Data Availability

The data presented in this study are available from the first author and the corresponding author upon reasonable request.
